# Impact of *Phytophthora* Disease on the Growth, Physiology and Ecosystem Services of Common Lime (*Tilia* × *europaea*) Street Trees

**DOI:** 10.1002/pei3.70054

**Published:** 2025-06-04

**Authors:** Eleanor Absalom, Anthony Turner, Matthew Clements, Holly Croft, Jill Edmondson

**Affiliations:** ^1^ Plants, Photosynthesis and Soils Cluster, School of Biosciences University of Sheffield Sheffield UK

**Keywords:** sap flow, tree diseases, urban cooling, urban forest

## Abstract

Tree diseases are a growing threat to ecosystem service provision by trees in cities and towns globally. *Phytophthora* is a widespread genus of plant pathogens (oomycetes) that have contributed to significant tree mortality worldwide; however, there has been little research into the impact of *Phytophthora* infection on urban trees or on ecosystem services important for urban populations, such as urban cooling. This study utilizes a network of Internet‐of‐Things linked sap flow sensors and point dendrometers collecting data every ~10 min throughout the growing season, combined with ground‐based sampling (leaf chlorophyll content, Leaf Area Index), to monitor the impact of *Phytophthora plurivora* on mature Common Lime ( *Tilia* × *europaea* ) street trees, a globally common urban tree species known to be susceptible to *Phytophthora*. *P. plurivora* infection disrupted tree water flux, with an 87% reduction in median diurnal water use in infected trees (24.84 (IQR 77.04) L tree^−1^ day^−1^) compared with asymptomatic trees (198.36 (IQR 88.22) L tree^−1^ day^−1^). Infection also significantly reduced stem growth, with median shrinkage in infected trees of −0.22% (IQR 0.32%) compared with 0.35% (IQR 0.20%) growth in asymptomatic trees over the study period (May–October). However, infected trees with less disease damage were able to maintain growth and urban cooling similar to asymptomatic trees during the study period, highlighting the tensions between controlling disease spread and public safety hazards while maintaining ecosystem service provision. Our research raises questions about the impact of *P. plurivora* on other critical ecosystem services and in other common urban tree species and settings.

## 1 Introduction

Urban forests provide many ecosystem services that benefit the urban population and the wider environment, including improving air quality (Grote et al. 2016), mitigating urban heat (Schwaab et al. 2021), and improving the health and wellbeing of urban citizens (Wolf et al. 2020). Varying according to species, large, healthy trees have the greatest capacity to provide ecosystem services (Hand and Doick 2019; Nowak and Aevermann 2019). Maintaining existing urban tree cover and planning future planting to ensure a diverse species selection is critical to maximize the benefits of urban forests (Sjöman and Östberg 2019). However, diseases caused by pests and pathogens present a major and growing threat, which in isolation, and in combination with other stressors (e.g., climate change or urban pollution), are resulting in increasing incidence of widespread urban tree mortality (e.g., Ordóñez and Duinker 2015; Lüttge and Buckeridge 2023; Referowska‐Chodak 2019; Raum et al. 2023). Several factors increase the risk and possible scale of disease outbreak in urban forests, and in street trees especially, including the increasing trade in plant material to meet tree planting targets (Hulbert et al. 2017; Sjöman and Östberg 2019); a typically low species and genera diversity (e.g., Yang et al. 2012; Stevenson et al. 2020; Galle et al. 2021); and linear, closely‐spaced planting and regular maintenance (e.g., pruning) (Webb et al. 2023). In temperate climates like the UK, climate change is projected to make conditions more favorable for pathogen establishment and dispersal, with milder and wetter winters (Frederickson‐Matika and Riddell 2021). Extreme summer heat and drought events will also exacerbate water stress, rendering urban trees more susceptible to infection (Tubby and Webber 2010; Contreras‐Cornejo et al. 2023).

*Phytophthora* (Oomycota) is a diverse, widespread genus of filamentous, osmotrophic eukaryotes containing more than 180 species and some of the most destructive plant pathogens in the world (Judelson and Blanco 2005; Lamour 2013; McGowan et al. 2020). *Phytophthora plurivora* is a soil‐borne root pathogen with a broad range of potential host tree species (Jung and Burgess 2009; Riddell et al. 2019; Linaldeddu et al. 2020; Taylor and Grünwald 2021; Green et al. 2021; Landa et al. 2021). Whilst originating in Asia, *P. plurivora* is now widespread in the UK and is commonly detected in soils in woodland environments, plant nurseries, and public and private gardens (Hulbert et al. 2017; Green et al. 2021). Common symptoms of *P. plurivora* infection include extensive fine root loss, bleeding bark lesions, root and collar rot, growth reduction, and eventually leaf chlorosis and crown dieback (Mrázková et al. 2010; Jankowiak et al. 2014; Jung et al. 2018; Linaldeddu et al. 2020; Taylor and Grünwald 2021). To date, *P. plurivora* has been implicated in the widespread decline of European beech ( *Fagus sylvatica* ), Oak (*Quercus spp*), and Alder (*Alnus spp*) species in European forests (Jung et al. 2013; Matsiakh et al. 2020), and foliage blight of ornamental and forest species in European and North American nurseries (Parke et al. 2014; Jung et al. 2016). However, few studies have examined the impacts of *P. plurivora* on urban tree growth and physiology or the resulting effect on the capacity of urban trees to provide ecosystem services (Cleary et al. 2017; Raum et al. 2023; Dale et al. 2022). Understanding the influence of *P. plurivora* on urban tree function is particularly important as many urban forests around the world are dominated by *Phytophthora* host tree species (e.g., Sjöman et al. 2012; Ma et al. 2020; Stevenson et al. 2020). Disease outbreaks could therefore have profound and long‐lasting impacts on urban forest composition and ecosystem service provision (Freer‐Smith and Webber 2017; Mitchell et al. 2019; Roebuck et al. 2022).

Ecosystem services of particular interest to urban tree managers and policy‐makers are the regulation of carbon and water fluxes by urban trees, due to their impact on climate change, urban cooling, and flooding (e.g., Pataki et al. 2021; Cavender‐Bares et al. 2022). Tree sap flow, the internal movement of water and nutrients through sapwood from tree roots to leaves, is an effective method for estimating tree water status and CO_2_ assimilation, and for modeling transpiration and evaporative cooling (Winbourne et al. 2020). Sap flow is strongly influenced by plant traits, such as size and water use efficiency (Helletsgruber et al. 2020), along with environmental conditions, including the atmospheric demand for water loss (e.g., vapor pressure deficit) (Grossiord et al. 2020) and water availability (e.g., Konarska et al. 2023). While several studies indicate that *P. plurivora* can disrupt sap flow and carbon uptake by inhibiting tree water and nutrient supply (e.g., Parke et al. 2007; Dinis et al. 2011; Vieites‐Blanco et al. 2023), none have so far quantified the impact on tree water use, carbon sequestration, or cooling in an urban setting. Measurement of radial stem dynamics can also indicate tree water status over diel cycles (e.g., Zweifel et al. 2021) and quantify the allocation of sequestered carbon to woody biomass over annual timescales (e.g., Simovic et al. 2024). However, few studies have so far measured short‐or long‐term radial stem dynamics in response to *Phytophthora* infection, and none in the context of the urban environment (Davison 2014; Colangelo et al. 2018; Milanović et al. 2020).

Here, we address this knowledge‐gap by monitoring the impact of a widespread pathogen, *P. plurivora*, on the health and function of street trees in a common urban tree species, common lime ( *Tilia* × *europaea* ), using Internet of Things‐linked sensors combined with ground‐based sampling. Smart sensors can offer a less labor‐intensive method to monitor disease progression in urban trees, enabling managers to make more rapid, informed choices (Dahlsjö 2023), but have so far rarely been utilized in tree disease research or practice (Potamitis et al. 2019). We aim to answer the following research questions:

1. How does *P. plurivora* infection affect tree morphological and physiological traits in *Tilia* × *europaea* street trees?
2. How does *P. plurivora* infection impact stem growth of *Tilia* × *europaea* street trees?
3. To what extent does *P. plurivora* infection impact water fluxes and the cooling benefits provided by *Tilia* × *europaea* street trees?

## 2 Materials and Methods

### 2.1 Site Description

**FIGURE 1:**
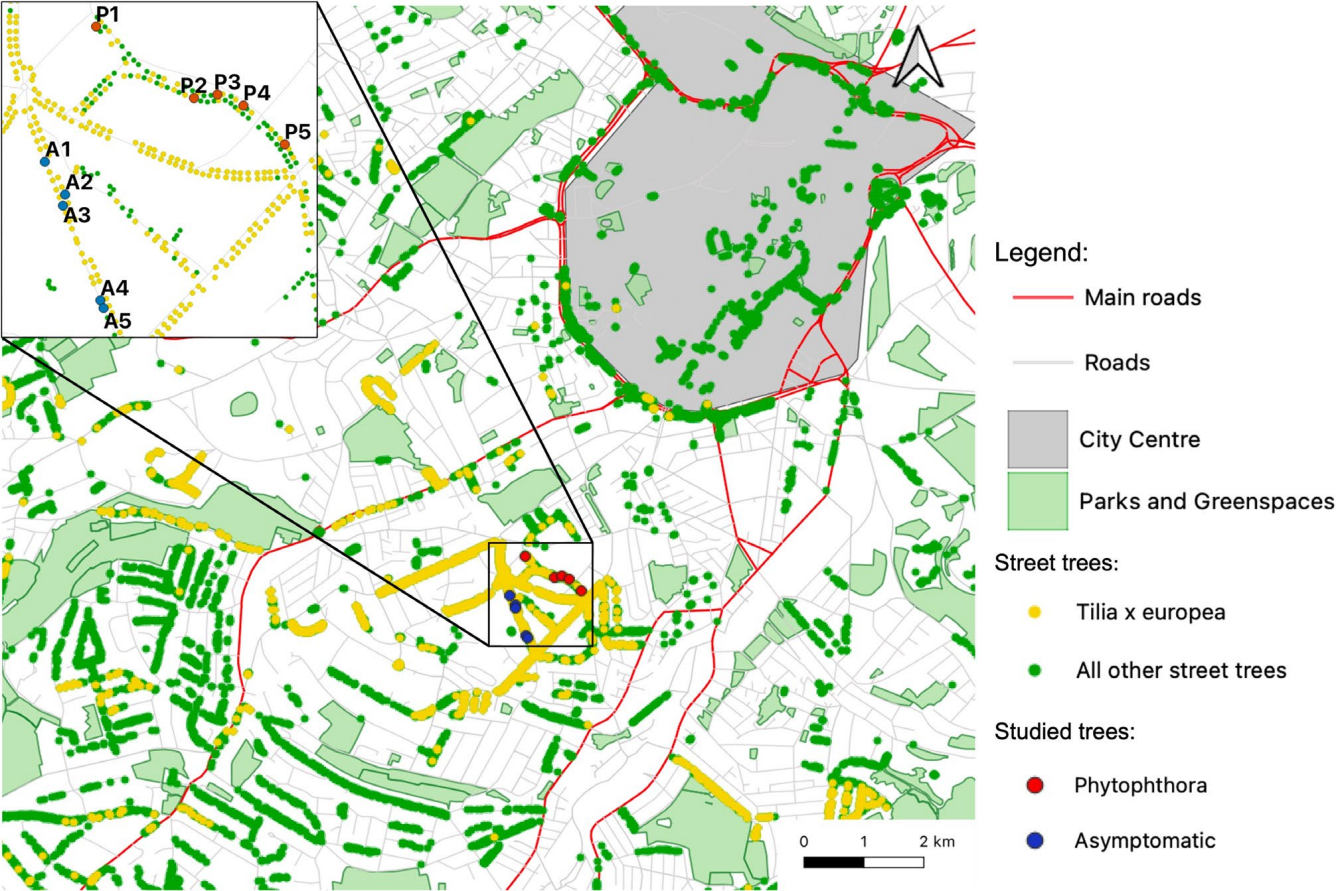
Location of asymptomatic and *Phytophthora plurivora* infected study trees within the city of Sheffield.

Ten mature *Tilia* × *europaea* trees on neighboring roads in a suburb of Sheffield, UK (53°22′ N, 1°28′ W), were monitored throughout May to September, 2022 (Figure 1). The roads are wide, residential streets with low traffic pressure and moderate housing and tree planting density. This area of Sheffield is dominated by *Tilia* avenues that were planted during the late 19th Century (Nether Edge History Society 2023). The selected trees are highly likely to be one of two cloned *Tilia* × *europaea* cultivars (‘Pallida’ or ‘Hatfield’), which comprise most *Tilia* plantings in the UK (Wolff et al. 2019).

**FIGURE 2:**
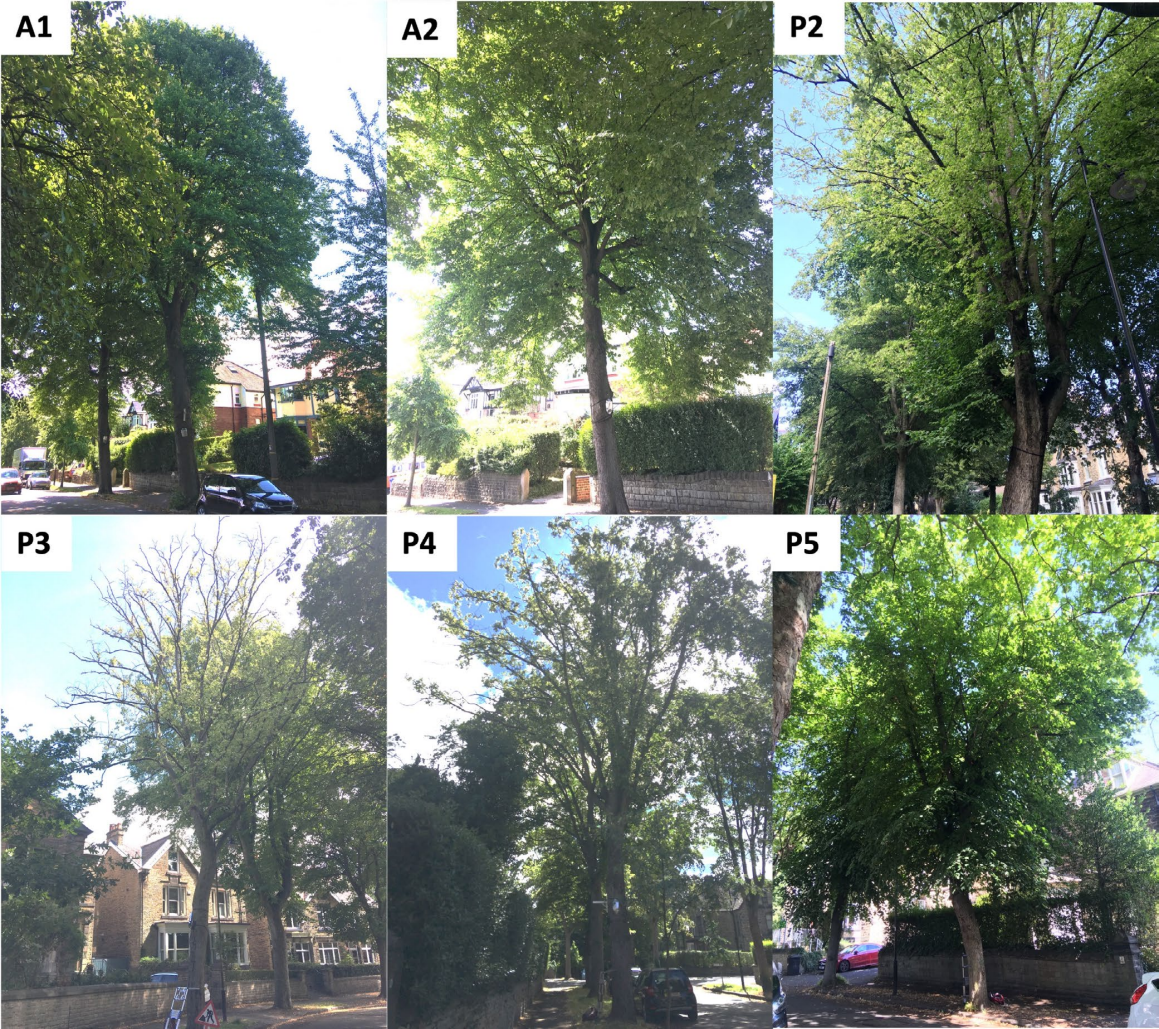
Images of some of the studied trees. A1 and A2: Asymptomatic trees; P2: Infected tree with some canopy dieback; P3 and P4: Infected trees with significant canopy dieback, leaf chlorosis and bleeding trunk lesions; P5: Infected tree with fewer visible symptoms.

Ten trees were selected—five with no visible signs of disease, hereafter referred to as asymptomatic A1‐5, and five with confirmed *P. plurivora* infection showing visible symptoms such as canopy dieback and sap bleeds, hereafter referred to as infected P1‐5 (Figures 1 and 2). Diameter at breast height (DBH) ranged from 47.5 to 78.7 cm and tree height from 17.14 to 19.11 m (Table S1), with no significant difference in median DBH or tree height between asymptomatic and infected trees. All trees were located in narrow (60–80 cm) grass verges, excluding A2 and P5, which were encased in built material (paving). *Phytophthora* infection was first confirmed in 2016 by the Forest Research Tree Health Diagnostic and Advisory Service from tissue samples of the infected study trees, with the species identified as *P. plurivora* in 2021. Symptoms were first noticed on other trees in the area in 2008, with some declining trees felled from 2014 to 2017.

There was a drought and three heatwave events during the study period, from June to August 2022, with the hottest day (39.4°C) on record occurring in Sheffield in July, exceeding the previous warmest day (in 2019) by 3.8°C (Barker et al. 2024). There was also 36% less precipitation between May and August 2022 (163.6 mm) in Sheffield than the long‐term (1990–2020) average (256.9 mm) (Met Office 2024).

### 2.2 Tree Morphological Measurements

Tree height, diameter at breast height (DBH) and average leaved‐canopy spread (m) were measured at the start of the study in May. Projected canopy area (CA) was derived according to:

$$\mathrm{CA}=\pi r^{2}$$

where *r* = average canopy spread in one direction.

### 2.3 Tree Sensors

Paired sap flow sensors and point dendrometers were installed at 3–3.5 m height on the north‐facing side of each tree between late March and early May 2022. The sap flow sensors measured sap flow every 60 min from 11th May to 30th September 2022, although sensors on trees A3 and P1 were damaged in late May and July, respectively, so only data prior to this was used for the following sap flow calculations for these trees. Point dendrometers measured trunk radial growth and air temperature every 15 min from 1st April or 9th May (depending on installation date) until 1st October 2022. Only data from May onwards was used for stem growth calculations to ensure consistency in the growth period between trees.

#### 2.3.1 Sap Flow Parameters

SF4 (UP GmbH Firmensitz, Germany) sap flow sensors measured sap‐flux density via the transient thermal dissipation method (Granier 1985). The temperature difference between two insulated, axially aligned 20 mm probes inserted into the xylem was measured every 10 min. Data was uploaded wirelessly via a LoRA network and processed using the PROSA application (https://logstar‐online.de/), with sap flux density calculated using the below equation (Granier and Gross 1987):

$$U=F_{G}\;{\left(\frac{∆T\max\left(\text{night}\right)}{∆T\text{actual}}-1\right)}^{\mathrm{EG}}$$

where *U* is the sap flux density in ml cm^−2^ min^−1^, ΔT_max (night)_ is the maximum temperature difference between probes during a period of minimal flow at night (between the hours of 8pm‐6am), Δ*T*_actual_ is the measured temperature difference between probes at the current time, Δ*T*_max(night)_/ΔT_actual_ is the dimensionless ratio between the two temperature differences, *F*_*G*_ is the Granier factor = 0.714 cm/min (=118.99·10^−6^ m/s) and *E*_*G*_ is the Granier exponent = 1.231 (dimensionless).

Whole‐tree sap flow in ml min^−1^ was calculated by multiplying the sap flux density by the sapwood area for each tree. As no cores could be taken, sapwood area was approximated according to the following equation (Meinzer et al. 2005; Schoppach et al. 2023):

$$\mathrm{SA}=a{\mathrm{DBH}}^{b}$$

where *SA* is the sapwood area in cm^2^, DBH in cm, and *a* and *b* are regression coefficients. While these are normally species‐specific, no values could be found in the literature for *Tilia* × *europaea* . Instead, coefficients derived from six diffuse‐porous sapwood species by Gebauer et al. (2008), including the closely related *Tilia cordata* and *Tilia platyphyllos* , were used (Ahongshangbam et al. 2023). Formulae devised by Rahman et al. (2017) in *Tilia cordata* street trees (mean DBH 45 cm) were used to account for the variation in sap flux density with sapwood depth (e.g., Gartner and Meinzer 2005; Rissanen et al. 2024).

Evaporative cooling by each tree was then calculated according to:

$$E=\mathrm{SF}\times L_{\mathrm{ev}}$$

where *E* is the energy loss per tree in Watts min^−1^, *SF* is whole tree water use in ml min^−1^, and $L_{\mathrm{ev}}$ is the latent heat of evaporation of water (2.45 kJ g^−1^) (Rahman et al. 2019). Tree water use and energy loss per minute were converted to *L* and kW, respectively, by dividing by 1000. Water use in *L* m^−2^ day^−1^ and energy loss in kW m^−2^ day^−1^ were calculated by dividing by the vertically projected canopy area of each tree and multiplying by 1440 (minutes in a day) (Konarska et al. 2023; 2016; Shashua‐Bar et al. 2023). Mean hourly and daily sap flow, water use and energy loss for each tree was then calculated across the study period, before deriving median hourly/daily values for all asymptomatic and infected trees.

#### 2.3.2 Radial Growth

Tree radial growth (μm) was measured using an automatic potentiometer‐based point dendrometer (TOMST, Prague, Czech Republic) (Matula et al. 2023; Yrttimaa et al. 2023). To improve data accuracy, loose outer bark was removed from the stem at the measurement area according to the manufacturer's recommendations. Data was downloaded manually using supplied software at the end of the study period.

Mean daily radial growth was calculated by averaging stem radius in μm for each tree over the study period, with stem radial growth change assumed to be symmetrical. Total stem radius change for each tree was calculated by subtracting the initial stem radius from the stem radius at the end of the study period. Change as a percentage of initial diameter was then calculated. Mean and median daily radial growth, total stem diameter change, and percentage diameter change were then calculated for each tree.

#### 2.3.3 Environmental Conditions

Air temperature was recorded by the point dendrometers every 15 min and an hourly average calculated, while hourly relative humidity was recorded by four additional tree sensors in the study area. Vapor pressure deficit (VPD), the difference between the amount of moisture in the air and the amount of moisture the air can hold at a given temperature, was calculated according to the following equation (Ouyang and Sun 2024):

$$\mathrm{VPD}=\left[1-\left(\frac{\mathrm{RH}}{100}\right)\times 610.7\times 10\;\frac{7.5\text{Temp}}{237.2+\text{Temp}}\right]$$

Where *VPD* is the vapor pressure deficit in Pascals (Pa), RH is relative humidity (%) and Temp is the air temperature (°C) as recorded by the tree sensors. *VPD* in kilo‐Pascals (kPa) was calculated by dividing VPD (in Pa) by 1000. Mean temperature, relative humidity, and *VPD* values were averaged over 24 h for the study period and across all asymptomatic and infected trees where possible.

### 2.4 Leaf Area Index

Effective leaf area index (LAI) (or foliage density) was measured in the middle of the growing season on 28th July 2022 between 11 and 2 pm using the LAI‐2200 (LI‐COR Biosciences, USA). This was performed according to the manufacturer's method for isolated trees, with pairs of above and below canopy readings taken in four compass directions (LI‐COR 2019). Due to smaller canopy clearings and to reduce disturbance by buildings, a 45° view cap was also used to enable above‐ground readings to be taken as close as possible to each tree. During sampling, sky conditions were cloud‐free and sunny, so a scattering correction was also taken and applied later using the FV2200 software package (LI‐COR 2019).

### 2.5 Leaf Chlorophyll Content

In the middle of the growing season, on 28th July 2022, eight sunlit leaves per tree were collected and immediately transported in cool dark conditions to the laboratory for the destructive measurement of leaf chlorophyll content. Three clips of known area per leaf were placed in 5 mL Dimethylformamide (DMF) and the chlorophyll was left to extract in solution for at least 1 week in the fridge. Absorbance was then read at 663.8 nm, 646.8 nm, and 480.0 nm using a Shimadzu spectrometer (Croft and Chen 2017). Leaf fresh weight was measured before drying the leaves in an 80°C oven for at least 48 h prior to measuring leaf dry weight.

### 2.6 Statistical Analysis

Statistical analysis was performed in R (version 2023.06.1 + 524). Due to the non‐normality of data, the Mann Whitney *U*‐test was used to compare median values of structural traits, leaf chlorophyll content, sap flow parameters, and stem diameter change between asymptomatic and infected trees. The interquartile range (IQR) is given to indicate the data variability. Spearman's correlation was used to determine the relationship between hourly sap flow and environmental measurements and between different measured traits across all trees.

## 3 Results

### 3.1 Tree Morphological and Physiological Traits

**FIGURE 3:**
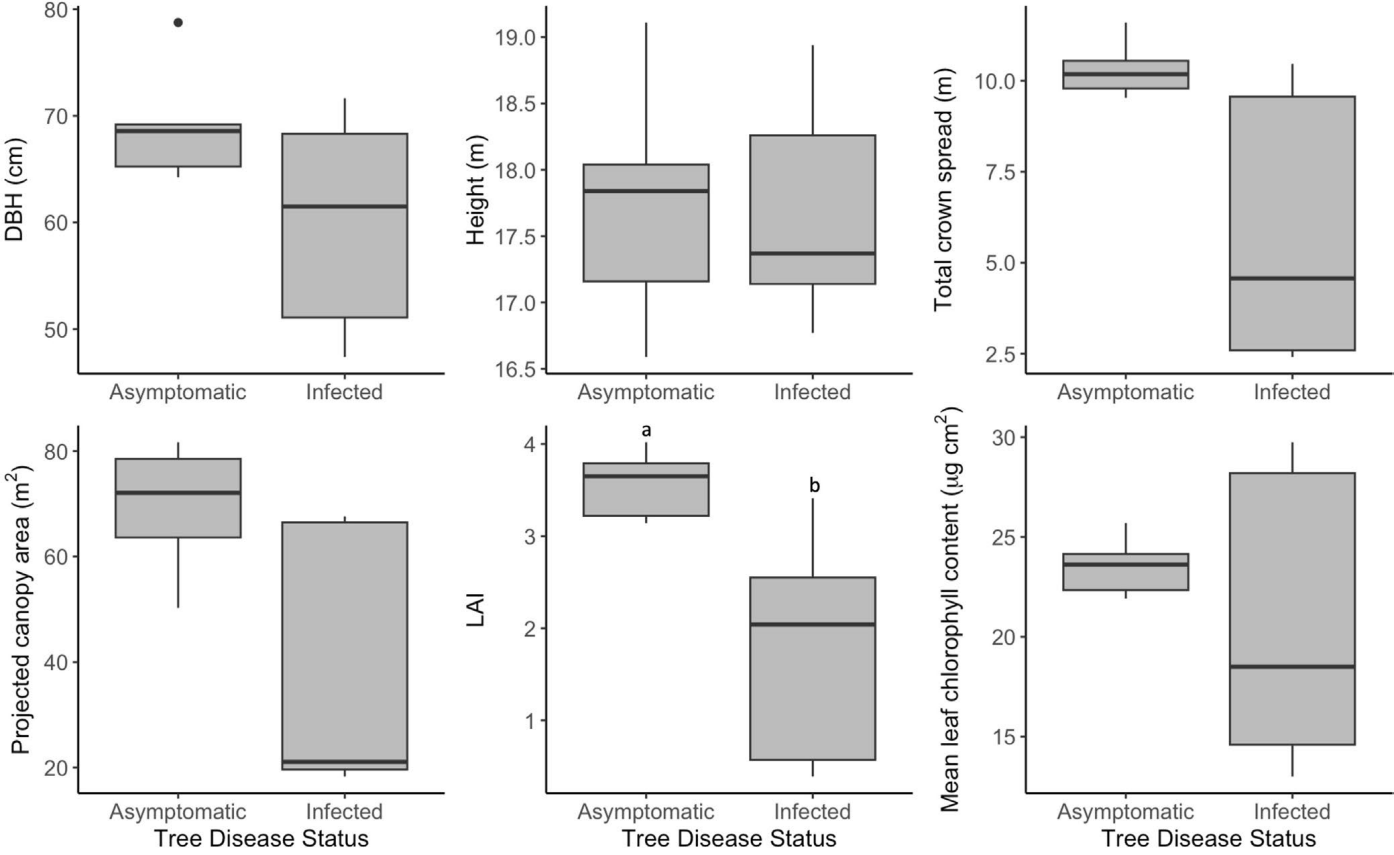
Structural and physiological traits of asymptomatic and infected trees. Different letters indicate a significant difference (Mann Whitney *U*‐test, *p* < 0.05) between median traits. Horizontal black lines indicate median, boxes show inter‐quartile range (IQR), whiskers show lowest and highest values within 1.5× IQR, and points denote outliers.

With the exception of tree height, all measured traits tend to be much more varied in infected trees compared with asymptomatic trees, and the median value is typically lower (Figure 3). However, there was no significant difference between median DBH, height, total canopy spread, or vertically projected canopy area in asymptomatic and infected trees, although the latter two traits were close to being significantly higher in asymptomatic trees (*p* = 0.09) (Figure 3) (full results in Table S1). Asymptomatic trees had significantly higher median Leaf Area Index (*U* = 23, n_1_ = 5, n_2_ = 5, *p* < 0.05) than infected trees, at 3.65 compared with 2.04. However, there was no significant difference between median leaf chlorophyll content of asymptomatic and infected trees (*p* > 0.05).

### 3.2 Seasonal and Diurnal Variations in Sap Flow

**FIGURE 4:**
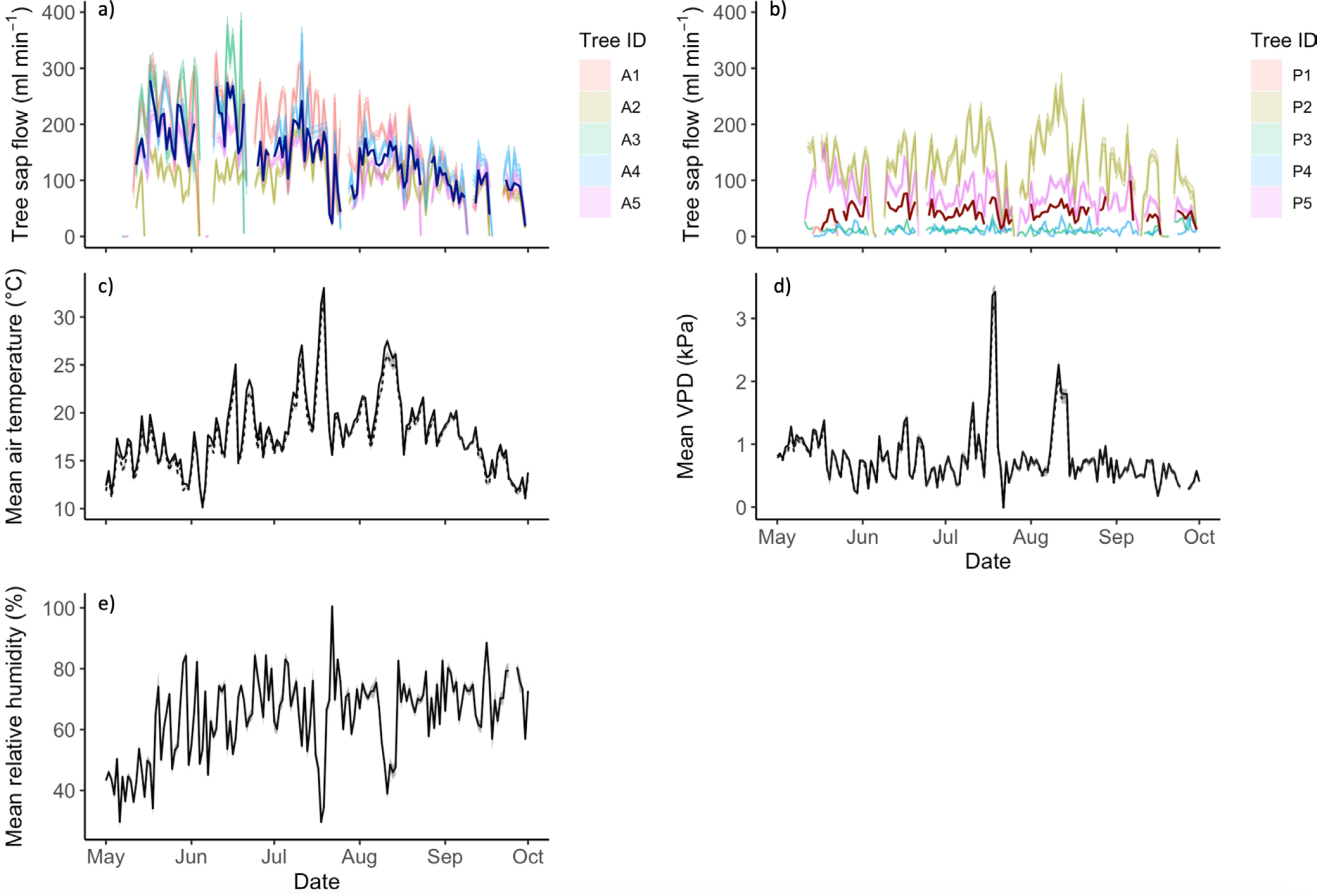
Diurnal sap flow for (a) asymptomatic trees and (b) infected trees, and (c) mean diurnal air temperature, (d) VPD and (e) relative humidity throughout the growing season. Faded lines show mean sap flow for each tree as detailed in figure legends, median for asymptomatic trees in dark blue, median for infected trees in dark red. Gaps are due to data loss (low battery or poor connectivity). Sap flow sensors on trees A3 and P1 were damaged during the study, hence missing data. Temperature data was collected from point dendrometers on each tree, with dashed line showing mean temperature and calculated VPD for infected trees, solid line showing mean for asymptomatic trees. Relative humidity was collected from additional tree sensors in the study area.

Daytime sap flow for all asymptomatic trees was highest in May and June (approximately ~130–400 mL min^−1^) and gradually declined over the growing season (Figure 4). Two of the infected trees (P2 and P5) had similar sap flow to asymptomatic trees in May, August (~100–250 mL min^−1^) and September (daytime mean: ~70 mL min^−1^) but showed a slight decline in peak summer (June and July) (daytime mean: ~90 mL min^−1^) (Figure 4). Mean sap flow during the night (Figure S1) was lower than during the day for all asymptomatic trees and the two aforementioned infected trees (night‐time mean: ~14–20 mL min^−1^). The remaining infected trees (P1, P3, and P4) had low, largely unchanging sap flow during the day and night and throughout the growing season (approximately ~8–20 mL min^−1^) (Figure 4). Across the whole growing season (May–September), median daytime sap flow was significantly higher in asymptomatic trees (133.22, IQR 87.25 mL min^−1^) compared with infected trees (27.06, IQR 82.27 mL min^−1^) (*U* = 23, n_1_ = 5, n_2_ = 5, *p* < 0.01). Median sap flow during the night was also significantly higher in asymptomatic trees (21.91, IQR 16.37 mL min^−1^) compared with infected trees (11.60, IQR 10.43 mL min^−1^) (*U* = 23, n_1_ = 5, n_2_ = 5, *p* < 0.01).

**FIGURE 5:**
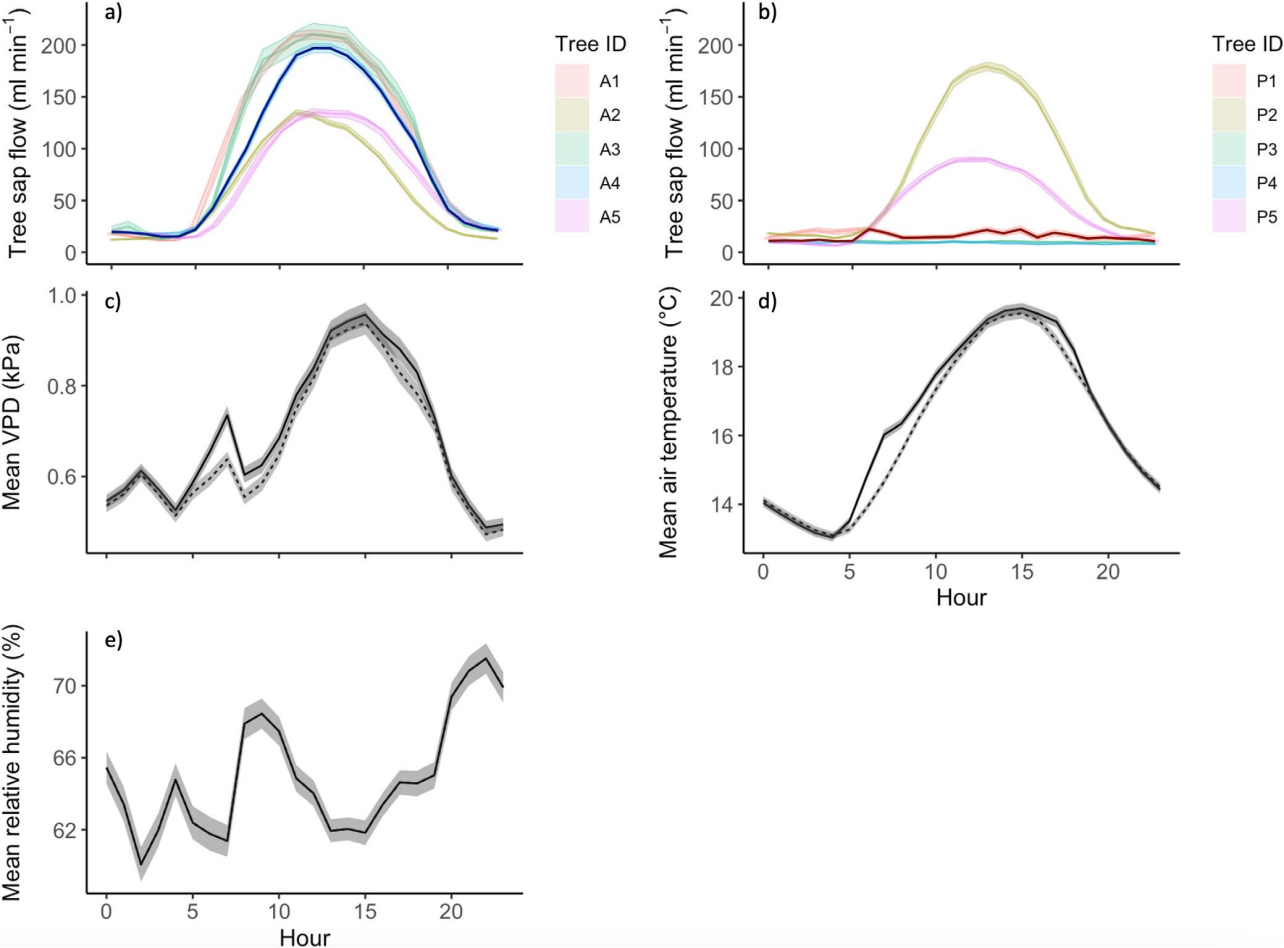
Mean hourly sap flow for (a) asymptomatic trees and (b) infected trees, and (c) mean hourly vapor pressure deficit, (d) air temperature, and (e) relative humidity. Faded lines show mean sap flow for each tree as detailed in figure legends, median for asymptomatic trees in dark blue and infected trees in dark red. Sap flow sensors on trees A3 and P1 were damaged during the study, hence missing data. Temperature data was collected from point dendrometers on each tree, with solid line showing mean temperature and calculated VPD for asymptomatic trees and dashed line for infected trees. Relative humidity was collected from additional tree sensors in the study area.

All asymptomatic trees and two infected trees (P2 and P5) exhibit a typical diurnal sap flow pattern, increasing in the morning, peaking and stabilizing during the middle of the day (approximately ~100–200 mL min^−1^), before declining overnight (Figure 5). In contrast, three of the infected trees (P1, P3, and P4) did not follow this pattern, with low and unchanging mean daily sap flow (Figure 5) (approximately 7–20 mL min^−1^).

**FIGURE 6:**
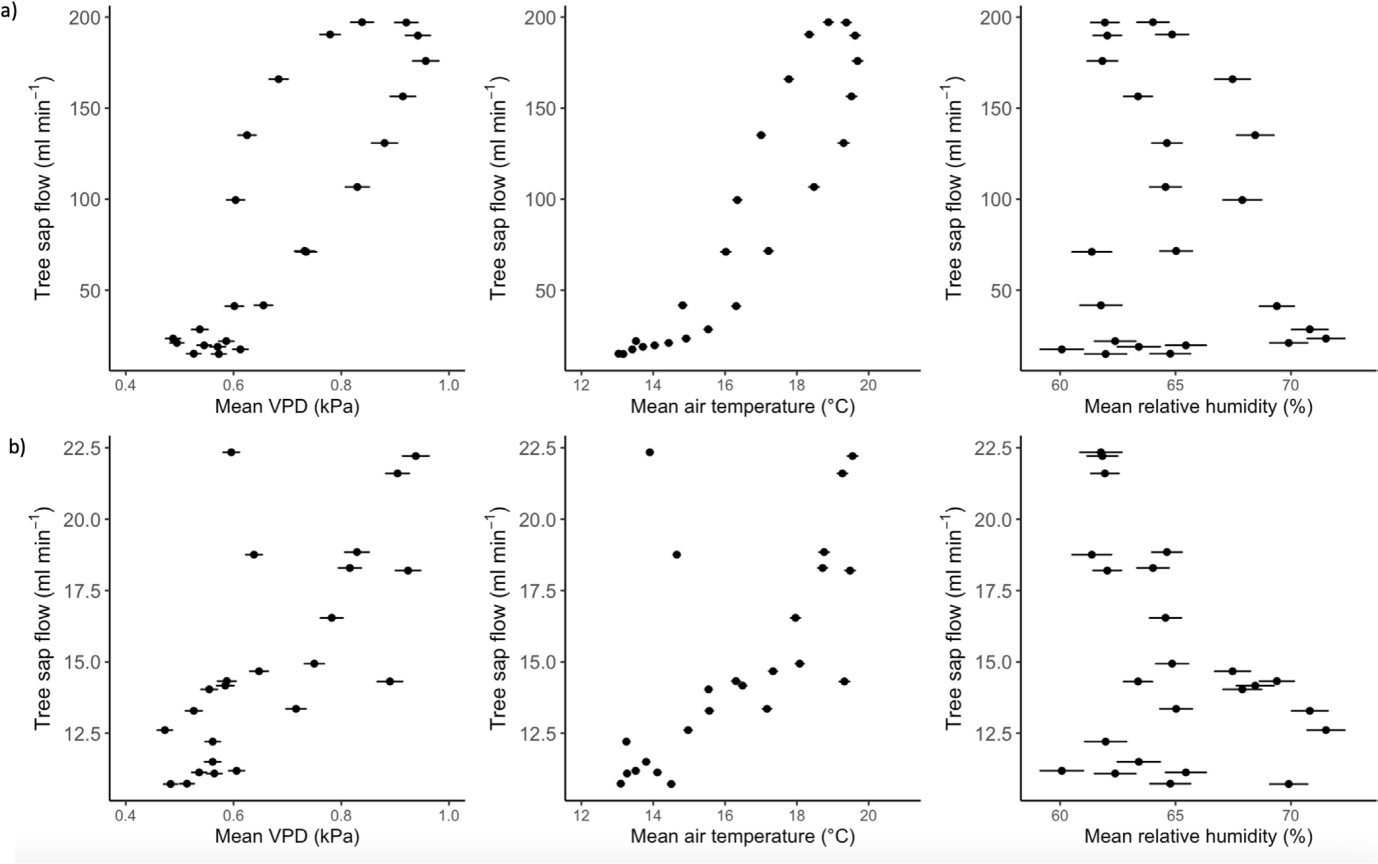
Median hourly sap flow and mean hourly vapor pressure deficit, temperature and relative humidity over 24 h during the growing season for a) asymptomatic trees (*n* = 5) and b) infected trees (*n* = 5). Bars show standard error of mean hourly climate variables.

Median hourly sap flow was strongly positively correlated with mean hourly air temperature (Spearman's rho = 0.93, *n* = 5, *p* < 0.001) and VPD (Spearman's rho = 0.83, *n* = 5, *p* < 0.001) (Figure 6a) in asymptomatic trees. There was a weaker correlation between median hourly sap flow and mean hourly VPD (Spearman's rho = 0.77, *n* = 5, *p* < 0.01) and air temperature (Spearman's rho = 0.70, *n* = 5, *p* < 0.01) in infected trees (Figure 6b). The sap flow of asymptomatic trees also displays a clear hysteretic pattern (time lag) in response to VPD and air temperature; however, this is not apparent in the infected trees (Figure 6). Interestingly, there was a trend of reduced sap flow with increased relative humidity in infected trees (Spearman's rho = −0.39, *n* = 5, *p* = 0.06), but not in asymptomatic trees (*p* > 0.05) (Figure 6).

### 3.3 Variations in Radial Stem Dynamics Across the Growing Season

**FIGURE 7:**
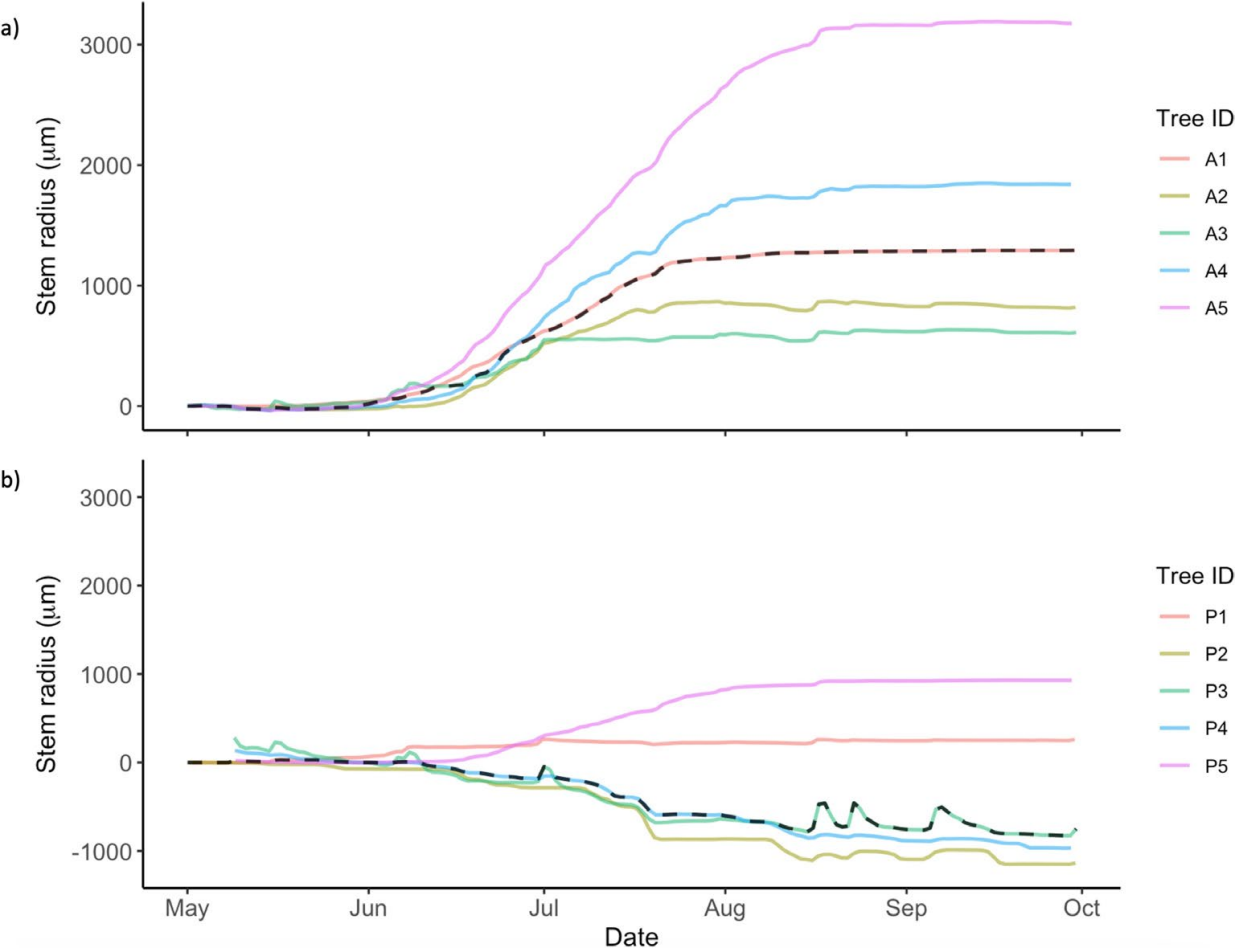
Stem radius (μm) of (a) asymptomatic and (b) *P. plurivora* infected trees over the study period, normalised to zero at the start of the study period in May. Faded lines show stem diameter change for each tree as detailed in figure legends, dashed line shows median.

Between April–October, tree stem diameter increased in all asymptomatic trees by a median value of 1196 μm (ranging from 728 to 3177 μm) (Figure 7a) (full results provided in Table S2). Stem diameter increased in two infected trees (P1 and P5) over the same period, by 192 and 864 μm respectively, while declining in the other three infected trees by 764–799 μm (Figure 7b) (full results provided in Table S2).

There was a significant difference between the change in median stem diameter across the growing season between asymptomatic (1196, IQR 977 μm) and infected (−764, IQR 973 μm) trees (Mann–Whitney *U*‐test: *U* = 23, n_1_ = 5, n_2_ = 5, *p* < 0.05). There was also a significant difference between the median percentage stem diameter change of asymptomatic and infected trees (Mann–Whitney *U*‐test: *U* = 25, n_1_ = 5, n_2_ = 5, *p* < 0.01). During the study period, the stem diameter of asymptomatic trees increased by a median of 0.35% (IQR 0.20%) of initial diameter (ranging from 0.22% to 0.93%), while the trunk diameter of infected trees decreased by a median of −0.22% (IQR 0.32%) of initial diameter (ranging from −0.30% to 0.36%) (full results provided in Table S2).

### 3.4 The Impact of *P. Plurivora* Infection on Ecosystem Service Provision

**FIGURE 8:**
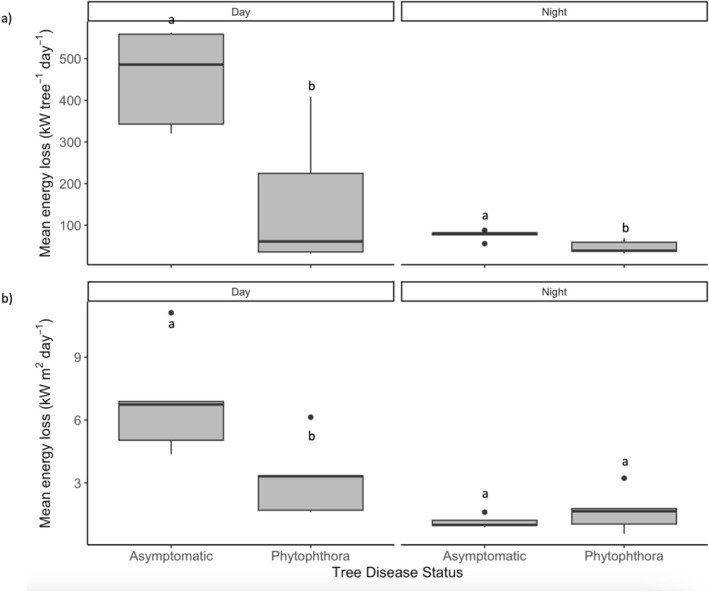
Daily energy loss (a) per tree and (b) per canopy area for asymptomatic and *P. plurivora* infected trees during the day and night. Different letters indicate a significant difference in median energy loss between infected and asymptomatic trees for each density and time period (Mann Whitney *U*‐test, *p* < 0.05). Horizontal black lines indicate median, boxes show inter‐quartile range (IQR), whiskers show lowest and highest values within 1.5 × IQR, and points denote outliers.

Across the growing season, median diurnal energy loss (indicating urban cooling through evapotranspiration) per tree is significantly lower in infected trees compared with asymptomatic trees, at 60.86 (IQR 188.74) kW tree^−1^ day^−1^ compared with 485.97 (IQR 216.13) kW tree^−1^ day^−1^ (Mann–Whitney *U*‐test: *U* = 23, n_1_ = 5, n_2_ = 5, *p* < 0.05) (Figure 8a). There was also a significant difference in median energy loss per tree overnight (Mann–Whitney *U*‐test: *U* = 23, n_1_ = 5, n_2_ = 5, *p* < 0.05), being higher in asymptomatic trees, at 80.58 (IQR 4.24) kW tree^−1^ day^−1^, compared with infected trees, at 38.62 (IQR 21.58) tree^−1^ day^−1^ (Figure 8a).

There was also a significant difference between median diurnal energy loss per canopy area (Mann–Whitney *U*‐test: *U* = 23, n_1_ = 5, n_2_ = 5, *p* < 0.05), with median energy loss in asymptomatic trees being higher at 6.74 (IQR 1.85) kW m^2^ day^−1^ compared with 3.32 (IQR 1.63) kW m^2^ day^−1^ in infected trees (Figure 8b). Interestingly, although there was no significant difference in median energy loss overnight (*p* > 0.05), infected trees had slightly higher energy loss per m^2^, at 1.65 (IQR 0.74) kW, than asymptomatic trees, at 0.99 (IQR 0.24) kW (Figure 8b).

The same trend is apparent for water use, given this is used to calculate energy loss. Per tree, median water use is significantly higher in asymptomatic trees than in infected trees during the day, at 198.36 (IQR 88.22) L tree^−1^ day^−1^ and 24.84 (IQR 77.04) L tree^−1^ day^−1^, respectively (Mann–Whitney *U*‐test: *U* = 23, n_1_ = 5, n_2_ = 5, *p* < 0.05). At night, the differences are smaller but remain significant, at 32.89 (IQR 1.73) L tree^−1^ day^−1^ and 15.76 (IQR 8.81) L tree^−1^ day^−1^ for asymptomatic trees and infected trees, respectively (Mann–Whitney *U*‐test: *U* = 23, n_1_ = 5, n_2_ = 5, *p* < 0.05; full results in Figure S2). Per canopy area, median water use during the day is significantly higher in asymptomatic compared with infected trees, at 2.75 (IQR 0.76) L m^2^ day^−1^ and 1.35 (IQR 0.67) L m^2^ day^−1^, respectively. There is no significant difference in water use per canopy area overnight (*p* > 0.05), although it is slightly higher in infected trees (median 0.67, IQR 0.30 L m^2^ day^−1^ compared with 0.40, IQR 0.09 L m^2^ day^−1^ for asymptomatic trees) (full results in Figure S2).

### 3.5 Correlation Between Tree Traits and Fluxes

**FIGURE 9:**
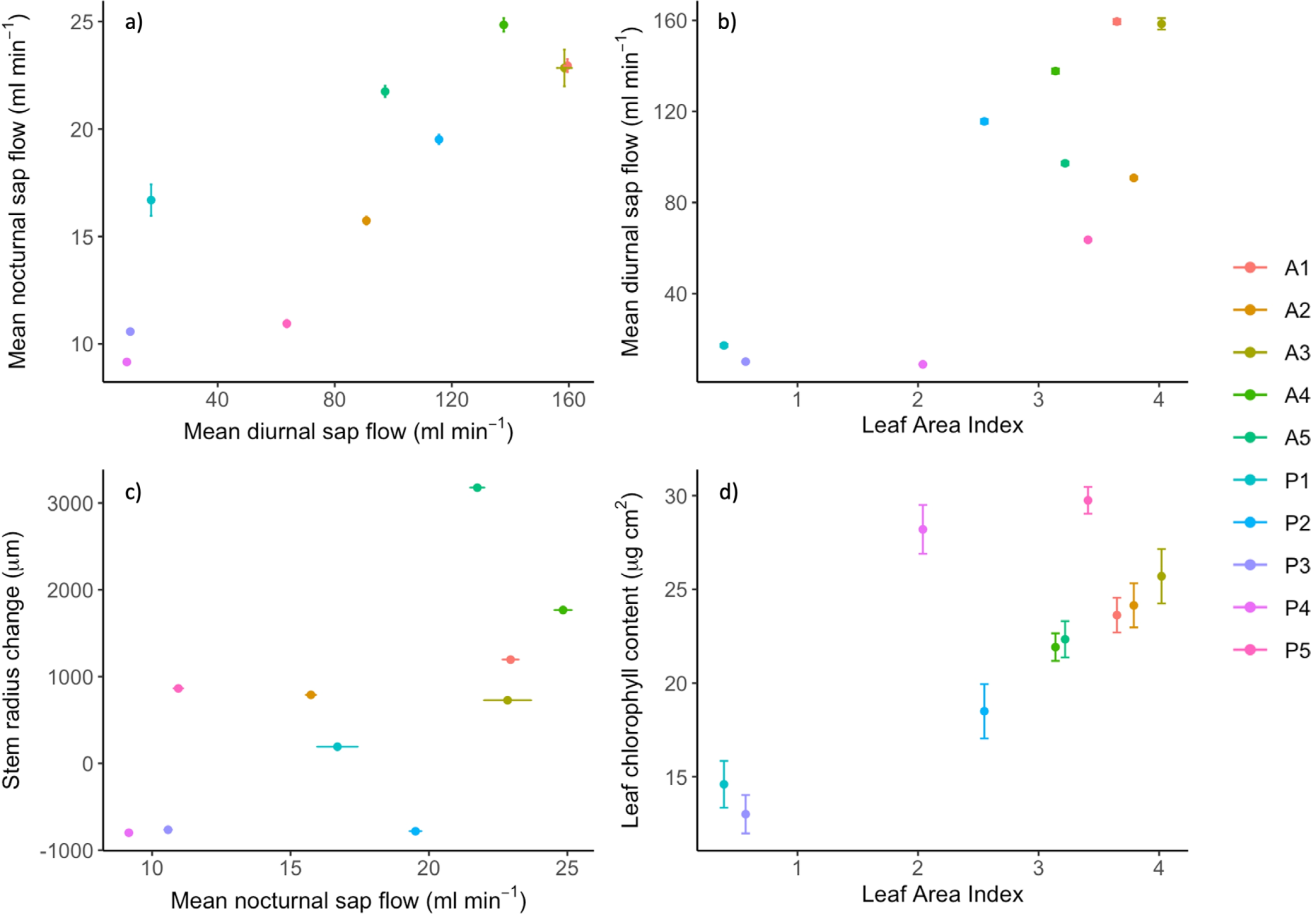
Correlation between traits and fluxes in asymptomatic and *P. plurivora* infected trees. Colors show each tree as detailed in the figure legend, bars show standard error of (a) mean diurnal sap flow (horizontal) and mean nocturnal sap flow (vertical), (b) mean diurnal sap flow, (c) mean nocturnal sap flow, (d) mean leaf chlorophyll content.

Across all trees, there is a strong, significant positive correlation between sap flow during the day and night (Spearman's rho = 0.92, *n* = 10, *p* < 0.001) (Figure 9a). Correlation between the other measured traits and fluxes is generally weaker, although it is significant between LAI and diurnal sap flow (Spearman's rho = 0.66, *n* = 10, *p* < 0.05), and between nocturnal sap flow and total stem radius change (Spearman's rho = 0.65, *n* = 10, *p* < 0.05) (Figure 9b,c). The correlation between LAI and leaf chlorophyll content is marginally significant (Spearman's rho = 0.62, *n* = 10, *p* = 0.06) (Figure 9d).

Generally, and as indicated by the significant positive correlations, the measured traits/fluxes tend to increase concurrently in the study trees. There are however some notable exceptions. For example, of the asymptomatic trees, A3 had the highest sap flow, leaf area index, and leaf chlorophyll content (day/night mean sap flow = 158.47/22.84 mL min^−1^, mean chlorophyll content = 25.70 μg cm^2^, LAI = 4.02), yet the lowest stem radial growth (728 μm, 0.23% of initial diameter) (Figure 9). Of the infected trees, P2 had similar sap flow and leaf area index as asymptomatic trees (day/night mean sap flow = 115.62/19.51 mL min^−1^, LAI = 2.55), but a large decline in stem radius (−781 μm, or −0.22% of initial diameter) (Figure 9), while P4 had similar leaf chlorophyll content and leaf area index as asymptomatic trees (mean chlorophyll content = 28.20 μg cm^2^, LAI = 2.04), but the lowest sap flow (day/night mean: ~9 mL min^−1^) and second largest stem radial shrinkage (−799 μm, or −0.26% of initial diameter) (Figure 9).

## 4 Discussion

This study investigated the impact of *P*. *plurivora* infection on the physiological and morphological traits, growth, and water fluxes of 10 established *Tilia* × *europaea* (common lime) street trees using Internet‐of‐Things sap flow sensors, point dendrometers, and ground‐based sampling. While the results should be interpreted with caution given the small sample size, the research provides valuable insights into the impact of *P. plurivora* on urban *T* × *europaea* trees and demonstrates the potential of Internet of Things‐linked sensors for urban tree disease monitoring.

Overall, we found that *P. plurivora* infection significantly suppressed trunk radial growth, LAI, sap flow, and urban cooling potential. In contrast, while crown dieback and reduced canopy area were observed in the infected trees, the trend was not significant, and the disease also had no significant effect on tree DBH, height, leaf chlorophyll content, or nocturnal water fluxes per canopy area. However, there was some variation in the extent to which the infected trees were impacted. Two trees (P1 and P3) had consistently lower values in all measured traits, while one (P5) had consistently high values that were similar to asymptomatic trees, with the remaining two trees displaying more mixed symptoms (Figure 9). We hypothesize that this variation is driven by the spread and stage of disease. Although the pathogenicity of *P. plurivora* in *T* × *europaea* is unknown due to a lack of studies in this tree species, tissue inoculation trials have shown a close relative, *T. cordata* , to have some resistance (Orlikowski et al. 2011; Cleary et al. 2017). Any genetic differences in disease resistance between the study trees are likely to be minimal given that they are cloned cultivars (Wolff et al. 2019) of a similar age. Furthermore, while disease spread and severity can be influenced by growth conditions and tree tolerance (Jung et al. 2018), the planting situation (e.g., density, Figure 2) and environmental conditions, such as air temperature and VPD (e.g., Figures 4 and 5), were analogous between study sites. As it can take several years of inoculum buildup and root destruction before above‐ground symptoms of *Phytophthora* infection, as measured here, show in mature trees (Erwin and Ribeiro 1996; Lamour 2013), it follows that the trees with more pronounced and consistent symptoms (P1 and P3) are at a more advanced stage of disease compared with the other study trees. However, we cannot confirm this theory as no measurements of pathogen density were taken in this study, and the exact date of infection for each tree is unknown. It is also possible that variation between trees in unmeasured growth conditions, such as soil quality, influenced host response and disease spread and severity (e.g., Jung et al. 2018).

The lack of a significant impact on most morphological and physiological traits in the tree canopy—tree height, canopy spread/vertically projected canopy area, and leaf chlorophyll content—likely reflects variation in disease severity (due to inherent tree resistance or stage of progression) in the infected trees. Root pathogens like *P. plurivora* tend to have fewer specific visible symptoms in tree crowns, resulting mostly from long‐term, secondary stressors, such as malnutrition and water deficit due to root and stem tissue damage (e.g., Orlikowski et al. 2011; Jung et al. 2018; Linaldeddu et al. 2020), increased herbivory (e.g., Milanović et al. 2015) or photosystem damage (e.g., Ďurkovič et al. 2021), rather than direct pathogen activity. Hence, it is typically the case that only trees with advanced infection display these symptoms (Lamour 2013). It may also explain the mixed symptoms in P4, which had similar LAI and chlorophyll content as asymptomatic trees but significantly suppressed sap flux and radial growth (Figures 3, 5 and 7). In contrast to related morphological traits (e.g., estimated canopy area), a statistically significant difference in LAI is likely because this measure can better account for heterogeneous canopy symptoms, such as canopy dieback, leaf chlorosis, and reductions in leaf size (e.g., Jung et al. 2018). Although the LAI‐2200 has been shown to underestimate LAI in single street trees (e.g., Klingberg et al. 2017), LAI estimates measured here correlate well with visual differences in leaf area (Figure 2), as well as measured canopy spread, vertically projected canopy area, and leaf chlorophyll content (Table S1). *P. plurivora* infection could therefore impact the urban cooling benefits provided by urban trees via shading, given that high LAI and canopy density are key traits for this (e.g., Rahman et al. 2020).

*P. plurivora* disease was associated with a significant reduction in tree water fluxes and stem radial growth, declining by approximately 87% and 164%, respectively. There was, however, variation in trunk diameter, water use, and energy loss in infected trees, with some trees showing clear deterioration in function, but others (P2 and P5) similar to asymptomatic trees. While *P. plurivora* infection also disrupted tree sap flow response to environmental drivers, there was similar variation. Overall, the correlation of median hourly sap flow with environmental variables that typically increase water loss through evapotranspiration, such as warmer temperatures and vapor pressure deficit (VPD) (e.g., Grossiord et al. 2020), is weaker in infected trees compared with asymptomatic trees (Figure 6), suggesting inhibition of sap flow. Furthermore, while asymptomatic trees show typical hysteresis in sap flow response to VPD and air temperature, a phenomenon documented in a range of tree species in natural and urban environments (e.g., Chen et al. 2011; Wan et al. 2023) and partially controlled by plant hydraulic properties (e.g., Zhang et al. 2014), infected trees do not (Figure 6). In contrast, the correlation with environmental variables that typically reduce water demand, such as high humidity (e.g., Cao et al. 2024), is higher in infected trees compared with asymptomatic trees (Figure 6). The impact of *P. plurivora* in disrupting tree sap flow response is particularly stark overnight, where nocturnal sap fluxes in three of the infected trees (P1, P3, and P4) are essentially unchanged from those during the day (Figures 4 and 8, Figure S2). Typically, sap flow declines overnight in response to reduced photosynthesis and atmospheric demand for evapotranspiration (Forster 2014), as in all asymptomatic and the remaining two infected trees (P2 and P5) (Figures 4 and 8, Figure S2).

Disrupted water and growth fluxes in P1, P3, and P4 are highly likely to be due to advanced *P. plurivora* infection, which has been shown to inflict significant damage to root and vascular tissues, including via root decay (e.g., Mrázková et al. 2010; Linaldeddu et al. 2020; Matsiakh et al. 2020), stem lesions, and collar rot (Orlikowski et al. 2011; Mrázková et al. 2013; Cleary et al. 2017; Haque et al. 2014; Jankowiak et al. 2014; Corcobado et al. 2020), destruction of phloem tissue (Clemenz et al. 2008; Ďurkovič et al. 2021), and occlusion of xylem vessels via pathogen hyphae and induced tyloses (Jung and Blaschke 1996; Parke et al. 2007; Brown and Brasier 2007; Vieites‐Blanco et al. 2023). Significant stem shrinkage in two of these trees (P3, P4) indicates that the impairment of root and stem functionality due to pathogen activity could also be leading to hydraulic failure (e.g., Davison 2014; Flower et al. 2018). This may have been exacerbated by the abnormally hot and dry conditions in the study year, with impaired response of these trees to increasing atmospheric water demand. Other research has, for example, found that *Phytophthora* infection can render trees more susceptible to drought (e.g., de Sampaio e Paiva Camilo‐Alves et al. 2013; Corcobado et al. 2014; Colangelo et al. 2018). In P2, which shows significant stem shrinkage but comparatively normal water fluxes and canopy traits, it may be that stem tissue damage is currently mild and/or largely confined to the phloem, with minimal xylem damage enabling relatively normal functionality to persist (e.g., Ďurkovič et al. 2021). The lack of a significant effect of *P. plurivora* infection on nocturnal energy loss and water use per canopy area is likely because the calculated canopy area of the infected trees, particularly those with more advanced dieback, tended to be smaller—for P1 and P3 it was only approximately a quarter of the projected area of the largest asymptomatic trees (~20m^2^ vs. 80m^2^), for example (Table S1)—while nocturnal water fluxes in more impacted trees tended to show little difference to those during the day (e.g., Figure 5). This would also explain their slightly higher estimates of water use and energy loss per canopy area overnight (Figure 8).

By inhibiting water transport, *P. plurivora* infection also imposes a significant reduction—approximately 87%—in the median transpirational urban cooling potential of *T* × *europaea* street trees (Figure 8). This is higher than has previously been measured in urban *T. europaea, T. cordata*, and other tree species due to hydraulic regulation in response to heat and drought events and urban conditions that restrict water availability, such as surface sealing (Rahman et al. 2017; Konarska et al. 2023). Changes in tree water fluxes will also impact the capacity of trees to sequester carbon. At the leaf‐level, there is a strong correlation between transpiration and CO_2_ assimilation (Chaves 1991), which can be scaled‐up using sap flow (i.e., whole tree transpiration) to indicate whole‐tree carbon assimilation (e.g., Köstner et al. 2008; Wang et al. 2014). Hence, reductions in sap flow, and thereby transpiration, for example due to water stress, have been shown to lead to reductions in CO_2_ assimilation (Hu et al. 2010; Zhang et al. 2019). Although our results show that the asymptomatic trees were not water stressed in the study year—maintaining high rates of sap flow despite isolated heat and drought events—other work in *Tilia* street trees has found a reduction in sap flow and transpiration in response to both similar and more extreme events (e.g., Rahman et al. 2017; Rötzer et al. 2021; Ahongshangbam et al. 2023). The synergistic effects of climate change and urban conditions could therefore exacerbate the negative impacts of *Phytophthora* infection on urban trees, including their capacity to regulate water and carbon fluxes, as well as rendering them more susceptible to infection (Werbin et al. 2020; Esperon‐Rodriguez et al. 2022). Despite this, little research has so far been conducted on the impact of tree disease on the ecosystem services, particularly urban cooling, provided by urban trees (Raum et al. 2023).

Overall, this research highlights that there are potential trade‐offs to consider in terms of management (i.e., tree removal) to limit the hazards from tree decline and further disease spread against the ecosystem services provided by well‐functioning diseased trees, particularly large‐stature, mature trees that have the greatest capacity to provide ecosystem services (Freer‐Smith and Webber 2017; Hand and Doick 2019; Dahlsjö 2023). More work on tree disease in the urban context is critical to improve our understanding of factors that may influence urban tree disease severity, and to inform sustainable urban forest management in response to tree disease outbreaks.

## 5 Conclusion

The results of this study have found that *P. plurivora* disease can significantly suppress morphological and physiological parameters in *Tilia* × *europaea* street trees, causing an 87% reduction in urban cooling. However, some trees, possibly at earlier stages of infection, are able to maintain physiology, growth, and ecosystem service benefits similar to asymptomatic trees. Given the growing threats of climate change and disease outbreaks in urban forests, further research is needed on the progression and impact of *Phytophthora* on other common urban tree species and important ecosystem services, particularly urban cooling. This will help to inform management and planting guidelines to create more resilient, sustainable urban forests.

## Conflicts of Interest

The authors declare no conflicts of interest.

## Supporting information


Data S1.

Table S1.
Figure S2.
Table S3.

## Data Availability

The data that supports the findings of this study are available in the supplementary material of this article.
