# Supplementary material for: Impact of Phytophthora Disease on the Growth, Physiology and Ecosystem Services of Common Lime (Tilia × europaea) Street Trees
Source: Plant Environ Interact. 2025 Jun 4;6(3):e70054. doi: 10.1002/pei3.70054 (PMC12136725; doi:10.1002/pei3.70054)
Supplement: Supplementary file 2 — Table S1. Figure S2. Table S3. [file PEI3-6-e70054-s002.docx]

| Tree ID | DBH (cm) | Height (m) | Canopy spread (m) | Vertically projected canopy area (m^2^) | Leaf Area Index | Mean leaf chlorophyll content (μg cm^-2^) |
| --- | --- | --- | --- | --- | --- | --- |
| A1 | 69.2 | 17.16 | 11.60 | 81.71 | **3.65*** | **23.62** |
| A2 | 64.1 | 18.04 | 9.53 | 63.62 | 3.79 | 24.14 |
| A3 | 65.3 | 16.59 | **10.18** | 50.27 | 4.02 | 25.70 |
| A4 | 78.7 | **17.84** | 10.55 | **72.08** | 3.14 | 21.92 |
| A5 | **68.6** | 19.11 | 9.79 | 78.54 | 3.22 | 22.34 |
| P1 | 68.3 | 18.94 | 2.41 | 18.28 | 1.39 | 14.60 |
| P2 | 71.7 | **17.37** | 9.56 | 66.48 | 3.55 | **18.50** |
| P3 | 51.1 | 18.26 | 2.59 | **21.06** | 1.57 | 13.00 |
| P4 | **61.5** | 17.14 | **4.57** | 19.63 | **2.04*** | 28.20 |
| P5 | 47.4 | 16.77 | 10.46 | 67.64 | 3.41 | 29.75 |

**Supplementary Materials**

**Supplementary Table S1.** Morphological and physiological traits of asymptomatic and *P. plurivora* infected trees. Values in bold indicate median for each group. DBH = diameter at breast height. Significant difference between median values indicated with * (Mann Whitney U-test, p<0.05).

*
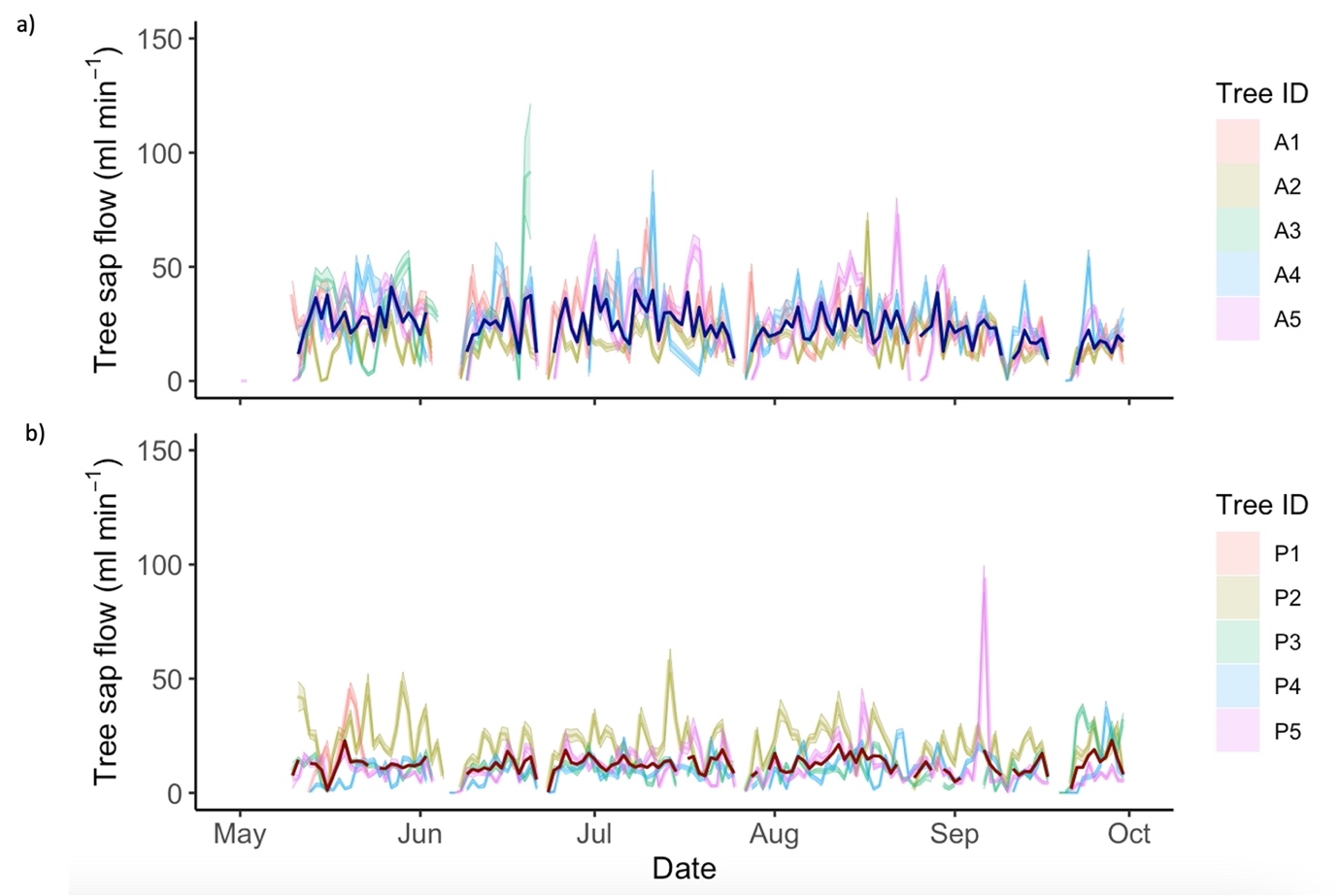
*

**Supplementary Figure S1.** Mean nocturnal sap flow of a) asymptomatic and b) *P. plurivora* infected trees throughout the study period. Faded lines show mean sap flow for each tree as detailed in figure legends, median for asymptomatic trees in dark blue, median for infected trees in dark red. Gaps are due to data loss (low battery or poor connectivity*).* Sap flow sensors on trees A3 and P1 were damaged during the study, hence missing data.

120.9 x 153.9mm (330 x 330 DPI)

| Tree ID | Disease status | Stem radius change (μm) | Stem diameter change (%) |
| --- | --- | --- | --- |
| A1 | Asymptomatic | **1196** | **0.35** |
| A2 | Asymptomatic | 790 | 0.25 |
| A3 | Asymptomatic | 728 | 0.22 |
| A4 | Asymptomatic | 1767 | 0.45 |
| A5 | Asymptomatic | 3177 | 0.93 |
| P1 | Phytophthora | 192 | 0.06 |
| P2 | Phytophthora | -781 | **-0.22** |
| P3 | Phytophthora | **-764** | -0.30 |
| P4 | Phytophthora | -799 | -0.26 |
| P5 | Phytophthora | 864 | 0.36 |

**Supplementary Table S2.** Total stem radius change in μm and as a percentage of initial diameter for all studied trees. Values in bold indicate median for each group.


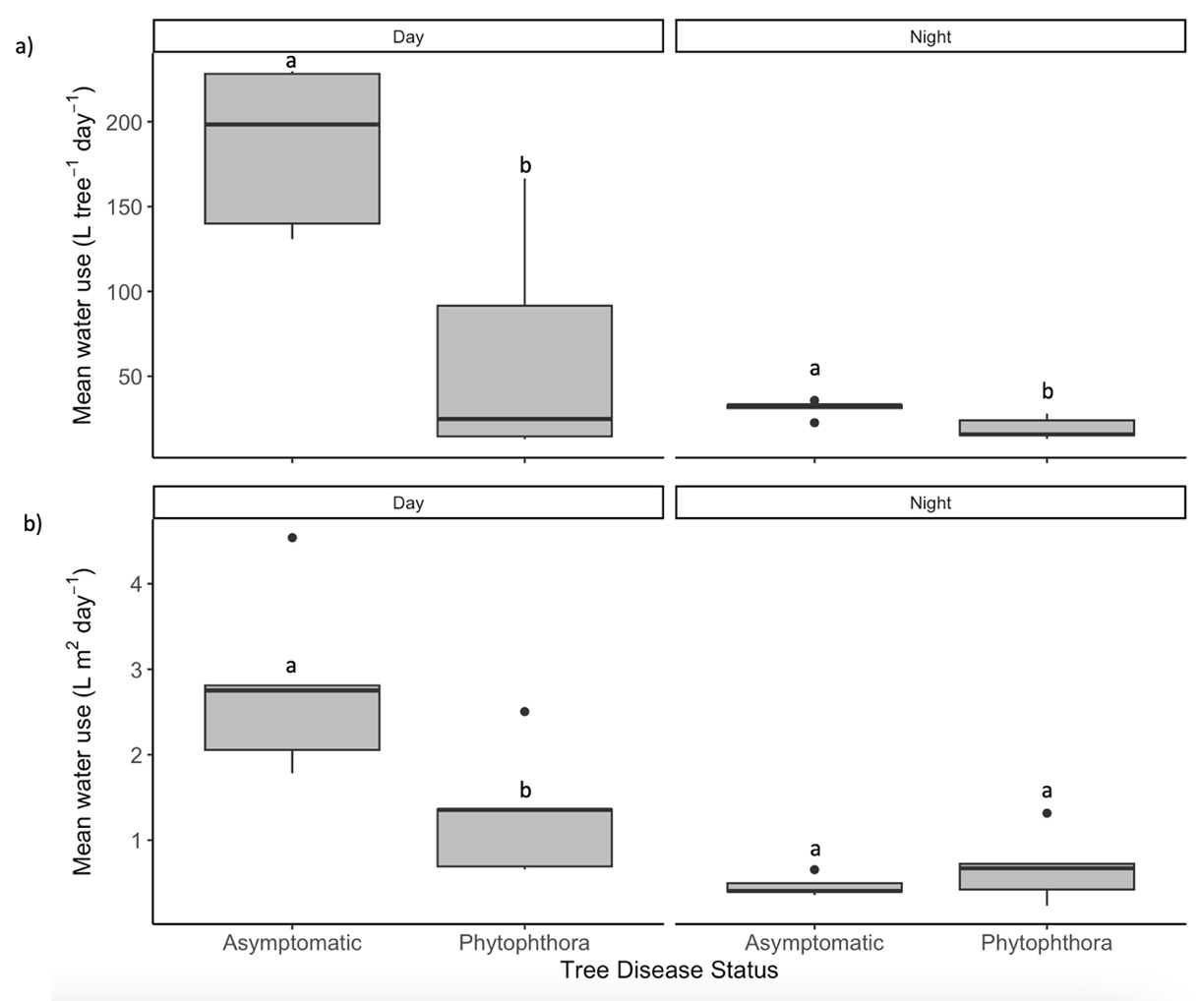


**Supplementary Figure S4.** Daily water use a) per tree and b) per canopy area for asymptomatic and *P. plurivora* infected trees during the day and night. Different letters indicate a significant difference in median water use between infected and asymptomatic trees for each density and time period (Mann Whitney U-test, p<0.05). Horizontal black lines indicate median, boxes show inter-quartile range (IQR), whiskers show lowest and highest values within 1.5 x IQR, and points denote outliers.
